# Synopsis of Remarks on Systemic Infection from Gonorrhœa

**Published:** 1891-12

**Authors:** J. Bedford Brown

**Affiliations:** Alexandria, Va.


### SYNOPSIS OF REMARKS ON SYSTEMIC INFECTION FROM GONORRHŒA.—BY J. BEDFORD BROWN, M. D., ALEXANDRIA, VA.—ILLUSTRATED BY CASES.

---

Dr. Brown, in his paper, cites five interesting cases of systemic poisoning from gonorrhœal infection. He believes that there are two channels for the absorption and conveyance or transmission of the gonorrhœal microbe into the general system. One is by continuity of surface over the mucous membrane of the genito-urinary tract from the urethra to the kidneys. The other channel is through the great lymphatic system, from the lymphatics of the urethra to the inguinal glands ; thence through the lymphatics of the system into the general circulation. He believes also that this microbe so transmitted is lodged at different points in the organism. The gonorrhœal microbe, transmitted by continuity of surface over the genito-urinary tract, invariably induces specific suppurative inflammation. On the contrary, when transmitted through the lymphatics the inflammation is not of a suppurative character, but assumes peculiar types. The contact of the infectious microbe with the mucous surfaces produces suppurative prostatitis, cystitis, ureteritis, pyelitis, then pyonephrosis. The absorption of the same through the lymphatic channels first sets up lymphangitis of the lymphatics of the urethra; then lymphadenitis of Cowper's glands; then of the inguinal glands; then inflammation of the connecting lymphatics. Then, by further absorption, it may induce septic phlebitis of the thigh, and finally synovitis. The question of systemic infection in the female from gonorrhœa through the uterus is omitted in this paper. He also believes that a genuine septic influence in certain cases of systemic poisoning, or in other words pyæmia, is developed, which his cases go to establish. He also believes that there is a marked relative difference in the susceptibility of different constitutions to the systemic poisoning of gonorrhœal infection, as in all other diseases, and that the infection of the system is only in certain cases.

The writer lays stress on gonorrhœal ureteritis as a part of the action of the gonorrhœal microbe in its travels over the mucous surface of the genito-urinary organs towards its final destination in this direction—the kidneys. It is accompanied with pain, at times sharp and paroxysmal, usually dull and creeping in character, along the trail of the ureter to the kidneys. These sharp paroxysms of pain extend upward to the kidneys, not downward as in nephritic colic. Then again there is soreness in the entire line of the ureter increased on pressure, so that the course of the canal can be marked out clearly. Ureteritis is always established before nephritis begins in gonorrhœal infection. The cases cited by Dr. Brown indicate that a state of pyæmia or septicæmia may be developed by the systemic infection from gonorrhœa in certain instances. Thus, he has seen septic infection from gonorrhœal prostat tis, cytitis pyonephrosis, lymphangitis and endocarditis.

CASE I.—A young man, aged about twenty-three, contracted gonorrhœa. He was previously in perfect health. The first two weeks his case progressed favorably, when a decided chill and fever ushered in acute prostatitis and then cystitis, ending in ureteritis and pyelitis, and finally pyonephrosis of the left kidney, with constant pain and tenderness over the region of the organ. Finally a large amount of pus appeared in the urine, which discharge of pus continued for three or four weeks. The urine, under the microscope, showed daily during all this suppurative stage as perfect and beautiful specimens of the malpighian bodies and uriniferous tubes as could be found in the most delicate anatomical preparations. Under a milk diet, bicarbonate of soda, quinine, the tincture of the chloride of iron, suppuration ceased and the man recovered with the loss of one kidney, and a closed ureter, but with perfect restoration of health.

CASE II.—A young married woman, aged about twenty-five, soon after marriage contracted gonorrhœa from her husband, but at the time was in a perfect state of health. The gonorrhœal infection extended from the ureter to the bladder, producing cystitis, then ureteritis, finally nephritis. The urine contained a large proportion of albumin. Both ureter and kidneys were involved in this case. These infectious inflammations were attended with great constitutional disturbances and depressions. There was continued fever, delirium, increasing stupor, then coma, and finally convulsions and death. There were all the symptoms present of septicæmia in the chills, fever and perspiration, the dry tongue, muttering delirium and coma. The history of this case, he thinks, suggests the thought that gonorrhœa may be more frequently the cause of nephritis, acute and chronic, than is usually supposed.

CASE. III.—The subject of this case, which he reports of an exceedingly interesting and remarkable character, was a young man of perfectly healthy character, aged twenty-seven. At about the tenth day of the attack of gonorrhœa symptoms of severe, acute prostatitis appeared, accompanied with violent pain in bladder and rectum and tenesmus, with frequent attempts at micturition. This was followed by complete retention of urine. It was impossible to introduce a catheter and aspiration was objected to. The urethra was douched by means of Davidson's syringe, with hot water containing cocaine, every half hour. This relieved the engorgement of the prostate and enabled the patient to void urine spontaneously. The inflamed prostate continued to give trouble, when the acidity of the urine reached a high point. A half ounce of bicarbonate of potash daily sufficed to keep the urine neutral, which always relieved prostatic irritation and pain. Under treatment the prostatitis gradually subsided. Then a prolonged rigor and violent fever ushered in a genuine attack of gonorrhœal cystitis, accompanied with discharge of blood and mucus. The urine now became alkaline and deposited an abundance of triple phosphates. As a local measure peroxide of hydrogen was thrown into the bladder through a soft catheter and thirty grains of benzoic acid in capsules were taken daily. On subsidence of the cystitis, ureteritis on both sides, denoted by pain and tenderness over the course of the ureters, came on; then desquamative nephritis, in which the urine contained albumin and casts and blood. Under the free use of benzoic acid, salol, iodide of potassa, the ureteritis and nephritis passed away, and the patient congratulated himself on his recovery after a confinement to bed for two months. In this he was disappointed. There was a slight return of ureteritis. Then there was lymphangitis in the lymphatics along the corpus spongiosum. Then the glands of Cowper became involved, and finally the inguinal glands. These glands became intensely swollen and inflamed, and the lymphatic vessels connected therewith became involved in the process, indicated by numerous red lines radiating over the groin and lower abdomen. There were also patches of erysipelatous inflammation. These local affections were accompanied with serious constitutional disturbance, as chills and fever. The lymphadenitis and lymphangitis were treated locally by means of an ointment containing iodine, iodoform and ichthyol. Internally ten grains of quinine and one grain of sulphide of calcium three times a day. The local affection subsided entirely under this treatment, but now the gonorrhœal infection localized itself on another structure.

In the femoral and saphenous veins a violent form of phlebitis appeared, preceded by chill and high fever. The veins became large, corded and painful. This complication was treated by the same ointment, quinine and tincture of iron, and ultimately passed away. But in a few days a most violent attack of gonorrhœal synovitis set in, heralded by chill and fever, involving the ankles, knees and hip joints. Under the phenacetine, salol and iodide of potash, the rheumatism subsided in about three weeks. This was followed by double orchitis, which was successfully treated by twenty grains of bromide of potassa, and five of the iodide every three hours. This was the last complication, and closed the history of the most extraordinary case of gonorrhœal poisoning on record. His patient was confined to the house about four months, and was in almost an exhausted condition.

CASE IV.—Male, aged 45, contracted gonorrhœa. Prostatitis ensued, with retention of urine. The urine was alkaline, but contained no albumin. Small abscesses formed in the prostate, symptoms of true septicæmia developed, consisting of chills and fever, and perspirations. The tongue became dry and brown, and there was complete loss of appetite, with nausea. Muttering delirium set in, then coma and death.

CASE V.—Male, aged 18, contracted gonorrhœa. In the third week severe gonorrhœal synovitis appeared in the joints of the arms and legs. Then septic endocarditis followed. With this there was high grade of fever, with adynamic or typhoid tendencies. Then, when convalescing from this complication, there suddenly appeared lancinating and intolerable pain in the center of the left eye, without conjunctivitis; then choroiditis and sclerotitis and loss of vision; then abscess and ulceration, and rupture of the cornea and destruction of vision. Iodide of potassium, the salicylates and alkalies exerted no effect whatever on the course of the disease.

The character of the disease was septic throughout. The writer believes that the microbe, when absorbed into the system through the lymphatics, is capable of assuming the character of a septic agent.

The writer believes that the gonorrhœal microbe, when absorbed into the system, either through the genito-urinary tract or lymphatics, wherever it finds lodgement, there it suspends or disturbs the process of sensation in the part, and sets up metabolic changes that induce inflammation, suppuration and destruction. Fortunately, the cases of its extension in absorption beyond the prostate glands or lymphatic glands of the groin are rare and exceptional.

